# Estimation of Corn Canopy Chlorophyll Content Using Derivative Spectra in the O_2_–A Absorption Band

**DOI:** 10.3389/fpls.2019.01047

**Published:** 2019-08-27

**Authors:** Xuehong Zhang, Yang He, Chao Wang, Fan Xu, Xinhui Li, Changwei Tan, Dongmei Chen, Guojie Wang, Lixin Shi

**Affiliations:** ^1^Key Laboratory of Meteorological Disaster, Ministry of Education (KLME), Joint International Research Laboratory of Climate and Environment Change (ILCEC), Collaborative Innovation Center on Forecast and Evaluation of Meteotological Disasters (CIC-FEMD), School of Remote Sensing & Geomatics Engineering,School of Electronic and Information Engineering, School of Geographical Sciences, Nanjing University of Information Science & Technology, Nanjing, China; ^2^Key Laboratory of Meteorology and Ecological Environment of Hebei Province, Meteorological Institute of Hebei Province, Shijiazhuang, China; ^3^Jiangsu Key Laboratory of Crop Genetics and Physiology, Jiangsu Co-Innovation Center for Modern Production Technology of Grain Crops, Joint International Research Laboratory of Agriculture and Agri-Product Safety of the Ministry of Education of China, Yangzhou University, Yangzhou, China; ^4^Department of Geography and Planning, Queen’s University, Kingston, ON, Canada

**Keywords:** corn, chlorophyll content, chlorophyll fluorescence, derivative spectra, O_2_–A absorption

## Abstract

Chlorophyll (Chl) is one of the most important classes of light-absorbing pigments in photosynthesis, and the proportion of Chl in leaves is closely related to vegetation nutrient status. Remote sensing-based estimation of Chl content holds great potential for evaluating crop growth status in agricultural management, precision farming and ecosystem monitoring. Recent studies have shown that steady-state fluorescence contributed up to 2% on the apparent reflectance in the 750-nm spectral region of plant and also provided additional evidence for fluorescence in-filling of the atmospheric oxygen absorption band at a central wavelength of 760 nm (O_2_–A band). In this study, an *in situ* hyperspectral remote sensing approach zwas employed to estimate corn Chl content at the canopy level by using chlorophyll fluorescence (ChlF) signals in the O_2_–A absorption band. Two new spectral indices, REArea_760_ (sum of first derivative reflectance between 755 and 763 nm) and REA_760_ (maximum of first derivative reflectance between 755 and 763 nm), derived from the first derivative spectra in the O_2_–A band, were proposed for estimating the corn canopy Chl content (CCC). They were compared with the performance of published indices measured at ground level, including the MERIS Terrestrial Chlorophyll Index (MTCI), Optimized Soil-Adjusted Vegetation Index 2 (OSAVI2), Modified Chlorophyll Absorption Ratio Index 2 (MCARI2), SR710, REArea (sum of first derivative reflectance between 680 and 780 nm), REA (maximum value of first derivative reflectance between 680 and 780 nm), and mND_705_. The results indicated that corn Chl content at the canopy level was better predicted by the new indices (with R^2^ = 0.835) than the published indices (with R^2^ ranging from 0.676 to 0.826). The two new indices ranked in the top four according to their summed ranks by integrating the ranks of RMSE and R^2^ of CCC linear regression models. ChlF originates only from chlorophyll in the photosynthetic apparatus and therefore is less sensitive to soil, wood, and dead biomass interference. Moreover, due to the fluorescence in-filling of the O_2_–A band and the amplified effect on spectrum signals by derivative operation, the spectral derivative indices in the O_2_–A band have great potential for estimating the CCC.

## Introduction

Photosynthesis, a chemical reaction converting light energy to chemical energy in glucose, is the basis for sustaining all plants’ life on Earth (Nelson and Yocum, 2006; Ustin et al., 2009). Chlorophylls (Chls) are vital light-absorbing pigments for photosynthesis (Gitelson et al., 2014), and thus their concentrations in leaves are related closely to primary production (Gitelson et al., 2006a; Houborg et al., 2013; Schull et al., 2015) and leaf nitrogen content (Clevers and Kooistra, 2012; Schlemmer et al., 2013; Kokaly and Skidmore, 2015; Ramoelo et al., 2015). Furthermore, leaf chlorophyll (Chl) content can be impacted by changes in plant type (Gitelson et al., 2006b), disease and nutritional and environmental stresses (Datt, 1999a), and plant phenology (Croft et al., 2014). Therefore, it is important to accurately estimate Chl content for agricultural management, precision farming and ecosystem monitoring.

There are two types of Chl existing in the photosystems of leaves: Chl a and b. Over the past few decades, studies have found that Chl *a* when extracted in diethyl ether has peak absorption wavelengths of 430 and 662 nm, while the peak absorptions of Chl *b* are at 453 and 642 nm (Du et al., 1998; Ustin et al., 2009). However, the strong absorption and weak penetrability to leaves near the peak absorption wavelength can result in the saturation of pigment absorption, which makes the reflectance spectra less sensitive to the Chl content at the wavelengths near the peak absorption band. (Gamon and Surfus, 1999). On the contrary, the spectral regions at the green and red edge region, ranging from 680 to 780 nm, have a strong penetrating power to leaves, and the reflectance spectra are highly sensitive to Chl content (Miller et al., 1990; Carter, 1998; Gupta et al., 2003; Sampson et al., 2003; Ustin et al., 2009). Therefore, the concentration of Chl within leaves can be estimated by measuring the absorption of light in the red and far red spectrum.

Previous research has indicated that vegetation Chl content can be retrieved using combinations of wavebands (i.e. vegetation indices) from remote sensing platforms. In remote sensing, canopy Chl content (CCC) is often used as a good indicator of canopy photosynthetic activity. CCC is defined as the product of the green leaf area index (LAI) and the leaf Chl content per unit leaf area. CCC can be derived from satellite observed signals by inversion of leaf optics and canopy reflectance physical modes as well as empirical models (Daughtry et al., 2000; Sims and Gamon, 2002; Wu et al., 2008; Gitelson et al., 2014). A number of spectral indices were proposed to estimate vegetation Chl contents, including i) reflectance-based indices (Dash and Curran, 2004; Reyniers et al., 2006; Wu et al., 2008; Zhu et al., 2008), ii) derivative-based indices (Filella and Peñuelas, 1994; Zarco-Tejada et al., 2003; Ju et al., 2010; Wei et al., 2013), and iii) feature-based indices (Boochs et al., 1990; Vogelman et al., 1993; Broge and Leblanc, 2001; Vincini et al., 2006).

Derivative-based indices have been widely used in monitoring vegetation with remotely sensed data. The reflectance spectra of vegetation are often characterized by an abrupt ascending slope in the “red edge” region, ranging from 680 to 780 nm (Horler et al., 1983), due to the strong absorption and scattering of incident solar radiation in the red and near infrared region. The biophysical properties of vegetation, canopy structure, atmospheric absorption and scattering, and soil backgrounds always affect canopy hyperspectral reflectance (Tsai and Philpot, 1998). However, derivative-based indices can minimize the influences of these background interferences and spectral noise and more effectively capture biophysical characteristics of vegetation from the canopy spectra (Li et al., 2013). Previous studies have indicated that canopy reflectance spectra in the red edge region can provide important information regarding biochemical composition and biophysical features (Datt, 1998; Sims and Gamon, 2002). Therefore, several indices calculated from the red edge reflectance and its first derivative reflectance, such as the red edge amplitude (REA: maximum derivative spectra in the red edge region), the red edge position (REP: defined as the wavelength of inflection point in the red edge region), and red edge area (REArea) are often used to estimate plant composition, such as crop Chl (Gitelson and Merzlyak, 1996; Sims and Gamon, 2002; Gitelson et al., 2003; Tang et al., 2004; Mutanga and Skidmore, 2007; Wei et al., 2013).

Some researchers found that REA was a good indicator of plant Chl content (Boochs et al., 1990; Tang et al., 2004; Ju et al., 2010). Other reports, however, found that the relationship between REA and plant Chl concentration was dependent on the vegetation types (Wang et al., 2003; Tang et al., 2004). These results implied that the relationship between REA and Chl content was poorly defined (Ju et al., 2010). REP, another major red edge parameter, shifted toward the longer wavelengths with increasing Chl content (Horler et al., 1983; Curran et al., 1990), and thus REP has been usually used to estimate Chl content (Wei et al., 2013; Li et al., 2017). But double-peak features of the vegetation derivative spectra weakened the usefulness of REP in monitoring Chl content (Li et al., 2017). Therefore, new or improved red edge spectral parameters are needed to improve the robustness and accuracy of assessing plant Chl content using hyperspectral data.

Previous studies also have found that Chlorophyll fluorescence (ChlF) is closely related to Chl *a+b* content (Ni et al., 2015; Wieneke et al., 2016). ChlF is the red- and far-red emission reemitted by the Chl molecules itself after light absorption (Porcar-Castell et al., 2014). Solar-induced ChlF emission spectrum is characterized by two peaks at approximately 690 and 740 nm (Meroni et al., 2009; Van der Tol et al., 2016). Typically, only about 1% of the absorbed sunlight is reemitted through ChlF (Baker, 2008) and contributed up to 2% on the apparent reflectance in the 750-nm spectral region (Campbell et al., 2002; Liu et al., 2005; Pérez-Priego et al., 2005). ChlF radiance at 760 nm (F_760_) generally increased with increasing Chl concentration while ChlF radiance at 685 nm (F_685_) decreased due to re-absorption of the emitted fluorescence signal, and the variations in the ratio of F_685_ and F_760_ were most likely related to structural variables such as CCC (Ač et al., 2015; Wieneke et al., 2016). Two main canopy parameters (Chl *a+b* content and LAI) have a considerable effect on the ChlF radiance in the atmospheric oxygen absorption band at the central wavelength of 760 nm (O_2_–A band), besides dry matter and the leaf inclination distribution (Ni et al., 2015; Van der Tol et al., 2016). Canopy fluorescence signal is not only related to plant photosynthetic capacity, but also varies with leaf and canopy biophysical and biochemical characteristics (Rossini et al., 2016). In addition, the evaluation of the fluorescence in-filling effects on reflectance showed that the apparent reflectance and its derivative reflectance in the 680-770nm range were sensitive to ChlF (Zarco-Tejada et al., 2003; Pérez-Priego et al., 2005; Ni et al., 2015).

In this paper, we have investigated the characteristics of derivative reflectance in the O_2_–A band for corn canopy. A novel remote sensing approach for estimation of corn Chl content using the derivative reflectance indices in the O_2_–A band has been developed and evaluated. Specifically, two objectives were addressed: 1) to evaluate the feasibility of estimating the CCC using two derivative reflectance indices: REArea_760_ (sum of the first derivative reflectance between 755nm and 763nm) and REA_760_ (maximum value of first derivative reflectance between 755 and 763 nm); and 2) to compare the proposed derivative reflectance indices to other chlorophyll-related vegetation indices used for estimating Chl content.

## Materials and Methods

### Experimental Design

**Figure 1:**
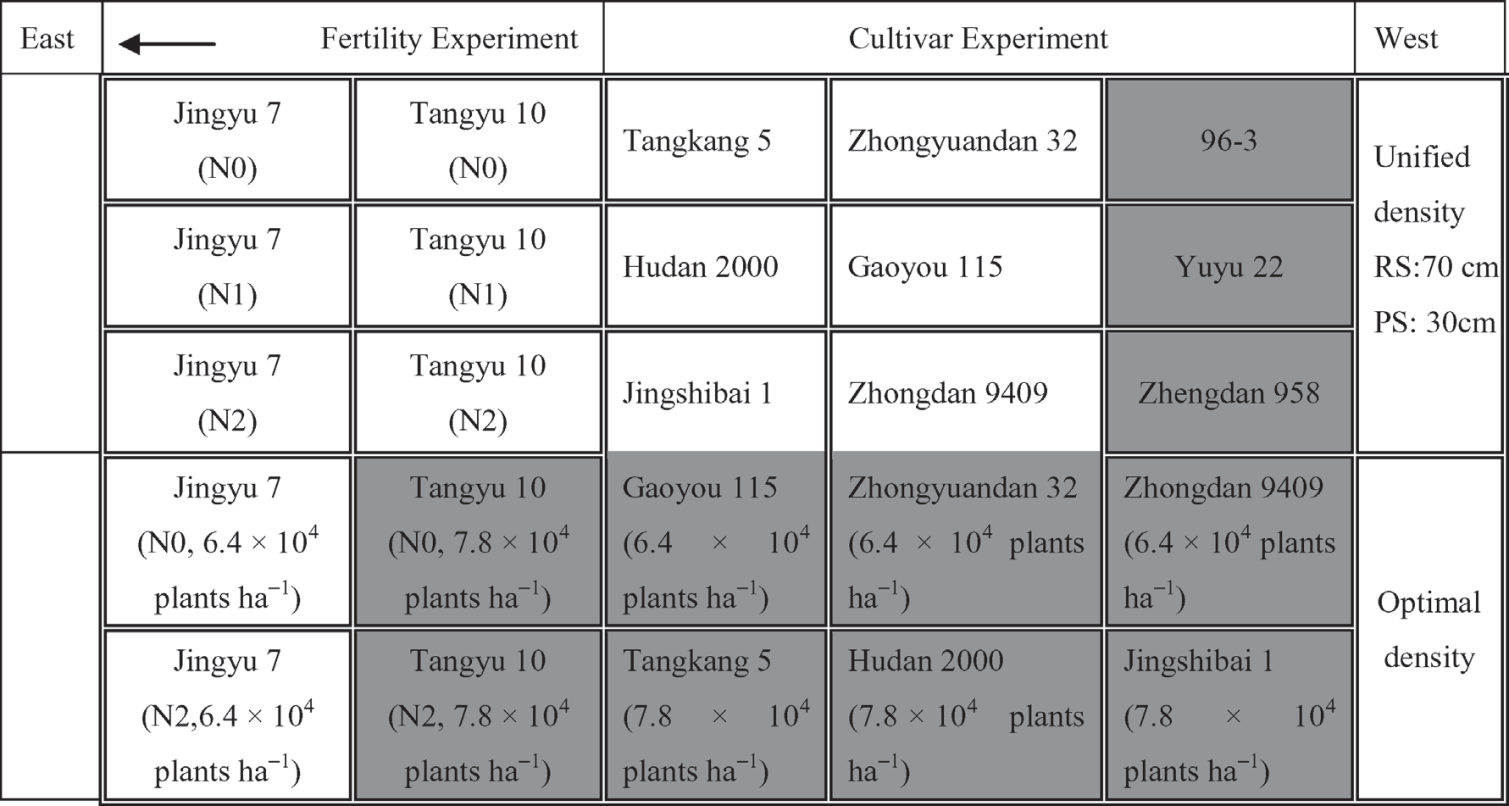
Field plot design in Exp.1 with different cultivars, density, and nitrogen fertilizer treatments. Eleven cultivars were selected, including four compact type cultivars, four semi-compact type cultivars, and three spread type cultivars. These were sown at a unified planting density with a row spacing of 70 cm and plant spacing of 30 cm. Two of these cultivars, i.e., Jingyu 7 and Tangyu 10, were selected to conduct a fertility experiment of the three nitrogen treatments. Moreover, 10 additional density treatment plots were also set up with a density of 7.8 × 10^4^ plants ha^−1^ for compact type cultivars and a density of 6.4 × 10^4^ plants ha^−1^ for semi-compact type cultivars. There were 25 15m x 7m plots in total. No plot had duplicated treatment with the others. N0, N1 and N2 represented N application treatments with the dose of 0, 75 and 150 kg ha^−1^, respectively. RS and PS denoted row spacing and plant spacing, respectively. The Chl content of the 11 shaded plots were measured only at VE and R1 growth stages.

Two field trials were conducted during the corn growing seasons of 2003. Experiment 1 (Exp.1) was carried out on a field with loam soil located at the Beijing Academy of Agriculture and Forestry Sciences, China (39°55′N, 116°16′ E). The field experiment included a fertility experiment and cultivar experiment. Eleven cultivars were sown, including four compact type cultivars (Tangyu 10, Hudan 2000, Jingshibai 1 and Tangkang 5), four semi-compact type cultivars (Jingyu 7, Zhongyuandan 32, Zhongdan 9409 and Gaoyou 115), and three spread type cultivars (96-3, Zhengdan 958 and Yuyu 22). Two of them, Jingyu 7 and Tangyu 10, were selected for a fertility experiment of the three different nitrogen treatments. In total, 15 plots were sown with a unified planting density of row spacing of 70 cm and plant spacing of 30 cm. In addition, 10 additional density treatment plots were also set up, with a density of 7.8 × 10^4^ plants ha^−1^ for compact type cultivars and a density of 6.4 × 10^4^ plants ha^−1^ for semi-compact type cultivars. The individual size of all 25 plots was 15 m × 7m. **Figure 1** shows the spatial distribution of all cultivars and nitrogen treatments of Exp.1. All the cultivars were sown on 20 June 2003 and each plot had no duplicated treatment with the others. Nitrogen treatments consisted of three doses [0, 75, 150Kg N ha^−1^] and the N applications were carried out on 15 July 2003 and 6 August 2003, respectively.

**Figure 2:**
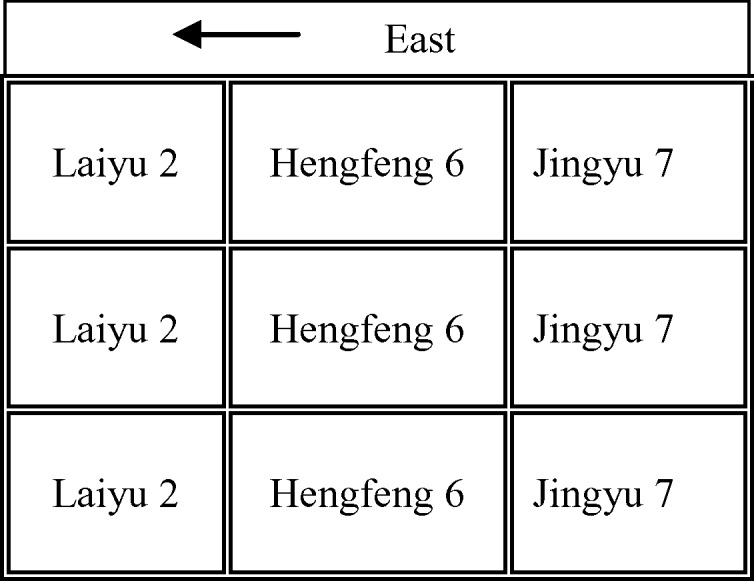
Field experiment design of Exp.2. Three corn cultivars (Laiyu 2, Hengfeng 6 and Jingyu 7) were sown at a density of 7.42 × 10^4^ plants ha^−1^ with three replicates. N was applied at 200kg N ha^−1^ for all corn cultivars.

Experiment 2 (Exp.2) was carried out on a field of 900 m^2^ (30 m ×30m) with silty loam soil at Luancheng experiment station located in Hebei Province, China (37°53′N, 114°41′ E). Corn was sown on 17 June 2003, in the north-south direction with 70cm row spacing and 30 cm planting spacing. Three corn cultivars (Laiyu 2, Hengfeng 6 and Jingyu 7) were sown at a density of 7.42 × 10^4^ plants ha^−1^ with three replicates. A total of 200kg N ha^−1^ was applied for all corn cultivars on 15 July 2003. **Figure 2** shows the spatial distribution of all cultivars in Exp.2. All nine plots were designed with a plot size of 9 m × 9m.

### Data Collection

#### Measurement of *In situ* Canopy Reflectance Spectrum

**Figure 3:**
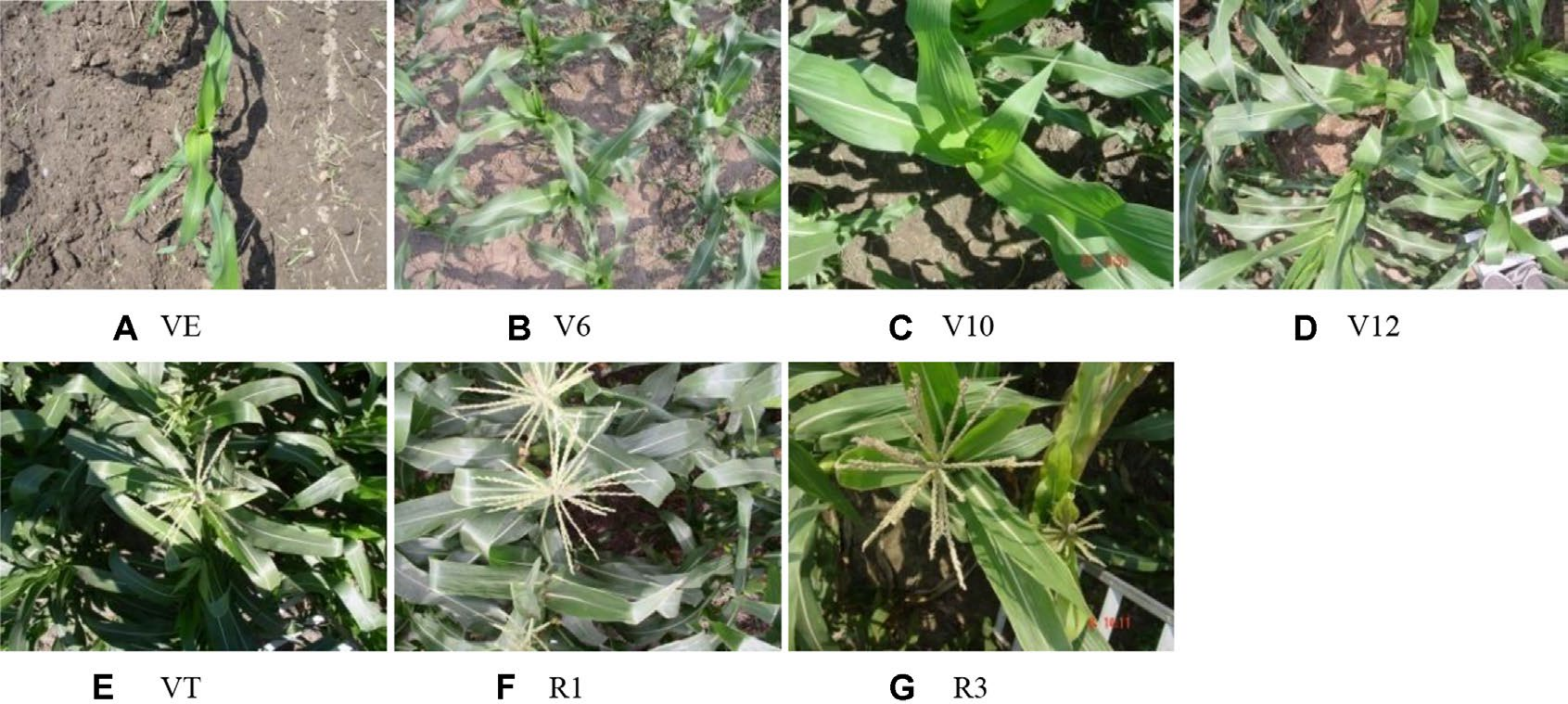
Photos taken from the top of corn canopy in Exp. 1 at VE **(A)**, V6 **(B)**, V10 **(C)**, V12 **(D)**, VT **(E)**, R1 **(F)**, and R3 **(G)** growth stages, illustrating the changes of the corn canopy with the growth stages.

The corn canopy reflectance spectra were measured by ASD FieldSpec Pro FR™ (Analytical Spectral Devices Inc., Boulder, Colorado, USA) fitted with 25° field-of-view fiber optics under clear sky conditions between 12:00–14:00 local time at seven growth stages: VE, V6, V10, V12, VT, R1, and R3. **Figure 3** exhibits the photos taken from the top of the corn canopy at different growth stages, illustrating the changes of the corn canopy with the growth stages. The fiber optics was fixed at the end of a horizontal pole carried by a tripod. This instrument recorded reflectance between 350 and 2500 nm with 3 nm and 10 nm resolution for the Ultraviolet/Visible-near infrared (UV/VNIR) (350–1000 nm) and shortwave infrared (SWIR) (1000–2500 nm) region, respectively. The hyperspectral data were re-sampled to 1 nm bandwidth using a self-driven interpolation method of the ASD spectrometer and then saved. Canopy reflectance spectral measurements were taken randomly at one site in Exp. 1 and three sites in Exp. 2 in each plot at a height of 1.6 m above plant canopy. Each spectral measurement was taken by averaging 20 scans at an optimized integration time with dark current correction. A 40 × 40 cm BaSO_4_ calibration panel was used to calculate reflectance at the canopy. The total numbers of canopy reflectance spectra acquired in Exp. 1 and Exp. 2 were 175 and 189, respectively.

#### Measurement of Canopy Chlorophyll Content

After each measurement of canopy spectral reflectance, five pieces of corn leaves from each site were chosen at random. From these leaf samples, the veins were removed and 0.2 g was cut from each sample. Chl was extracted in 90% acetone. Absorption was measured at 470, 649 and 665 nm with the spectrophotometer. The average value of three replicates was used to calculate the content (Chl_F_, mg/g fresh leaf mass) of leaf Chl *a* and Chl *b* using the formula described by Lichtenthaler (1987). In addition, for each sample, all green leaves were separated from stems and destructively sampled for leaf fresh weight (W_F_) measured using a Sartorius CPA324S electronic balance. Finally, these samples were oven-dried at 105°C for 30 min to destroy tissues and then dried at 80°C until a constant dry weight was reached (dry weight, W_D_) (Jing et al., 2007; Feng et al., 2017).

Leaf water content (LWC) was calculated as follows:

(1) $$\text{LWC}=\left({\text{W}}_{\text{F}}-{\text{W}}_{\text{D}}\right)/{\text{W}}_{\text{D}}\times 100\%$$

Leaf Chl content for dry leaf (Chl_D_, mg/g dry leaf mass) was estimated as follows:

(2) $${\text{Chl}}_{\text{D}}={\text{Chl}}_{\text{F}}/\left(1-\text{LWC}\right)\times 100\%$$

The LAI was measured using a destructive sampling method. All the corn plants within an area of 100 cm × 100 cm were sampled in the laboratory using the specific leaf weight (SLW) method (Bréda, 2003).

Total canopy chlorophyll content (CCC) at each growth stage was estimated as follows:

(3) $$\text{CCC}={\text{Ch}}_{\text{D}}\times \text{SLW}\times {\text{LAI}}_{\text{green}}\times 0.01$$

where the unit of CCC is g/m^2^. LAI_green_ represents the green LAI and SLW is the ratio of dry mass to leaf area (mg/cm^2^).

CCC data were estimated for 120 groups in Exp. 1 and 178 groups in Exp. 2 using the above method.

### Data Analysis

#### Construction of Derivative Spectral Indices in the O_2_–A Absorption Band

**Figure 4:**
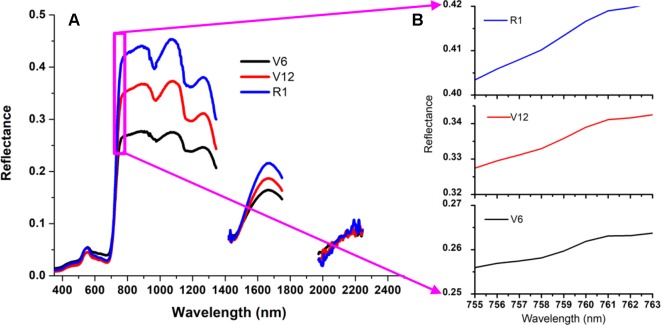
Three canopy reflectance spectra measured at the V6, V12 and R1 growth stages for the Jingyu 7 cultivar from Exp. 1 **(A)**. These apparent reflectance spectra ranging from 755 nm to 763 nm were demonstrated by local enlarged **(B)** and characterized by a weak peak at 761 nm because of the in-filling effects in the O2–A absorption band.

**Figure 5:**
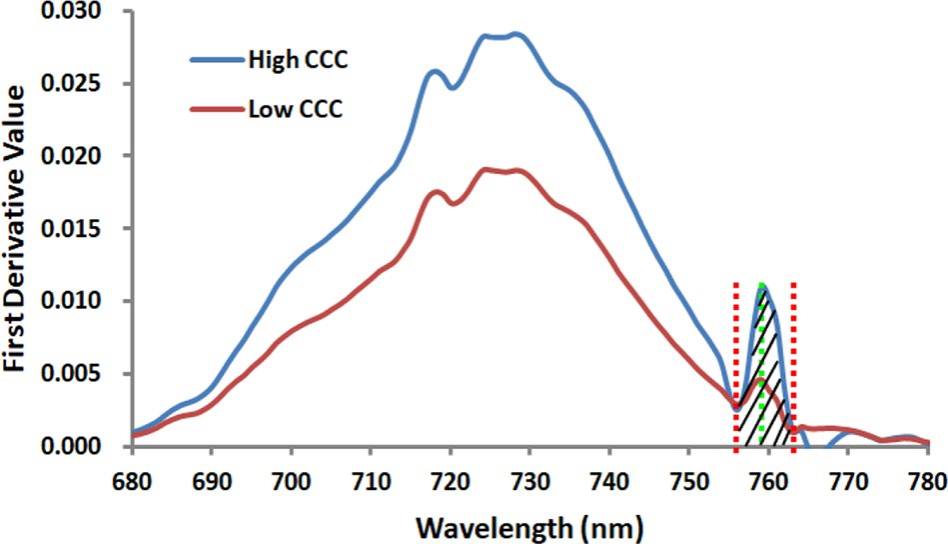
The curves of two proposed first derivative spectral indices REArea_760_ and REA_760_ for the Jingyu 7 cultivar measured at V6 and V10 growth stages in Exp.1. The high CCC and low CCC were 3.58 and 0.91g/m^2^, respectively. REArea_760_ is defined as the sum of the first derivative reflectance ranging from 755 nm to 763 nm, and REA_760_ is the maximal among the first derivative value from 755 nm to 763 nm.

There is a weak peak at 761 nm for the apparent reflectance (**Figure 4**) due to the solar-induced ChlF emission and the in-filling effects in the O_2_–A absorption band. Therefore, this unique feature can give rise to a significant peak for the first derivative spectra ranging from 755 nm to 763 nm (**Figure 5**). The ChlF in-filling in the O_2_–A absorption band at 760 nm was sensitive to variations of fluorescence (Pérez-Priego et al., 2005), and the Chl *a+b* content and LAI were highly correlated with F_760_ (Ni et al., 2015; Van der Tol et al., 2016). Accordingly, this study was intended to examine the feasibility of estimating crop CCC by developing two new indices, i.e., REArea_760_ (sum of the first derivative reflectance between 755nm and 763nm) and REA_760_ (maximum value of first derivative reflectance between 755 and 763 nm).

REArea_760_ is defined as:

(4) $${\mathrm{REArea}}_{760}=\overset{763}{\underset{755}{\int }}\frac{{\mathrm{dR}}_{\lambda }}{d\lambda }d\lambda$$

REA_760_ is denoted as:

(5) $${\mathrm{REA}}_{760}=\max\left({\mathrm{dR}}_{755}:{\mathrm{dR}}_{763}\right)$$

#### Model Calibration and Validation

Correlation analyses were performed between the spectral indices related to Chl content and corn CCC using SPSS 17.0 (SPSS, Chicago, IL, USA). In total, 30 existing spectral parameters were calculated (**Table 1**), and linear inversion models for CCC were established based on Exp.1. The coefficient of determination (R^2^) was used to evaluate these models. Additionally, to investigate the robustness of the spectral indices, we employed the data from Exp.2 to validate the fitted linear inversion models based on the data from Exp.1. The predictive performance of the spectral indices was assessed by ranking the RMSE values in ascending order. The overall performance of the spectral indices was then evaluated by finding the sum of the RMSE ranks and the R^2^ ranks of fitted linear inversion models. Finally the spectral indices were ordered according to their summed ranks, such that the best performing spectral indices had the lowest summed rank. Root mean square error (RMSE) in the equation were utilized to measure the fitness between predicted and observed values. RMSE was calculated with the following formula:

(6) $$\mathrm{RMSE}=\sqrt{\frac{1}{n}\times \overset{\text{n}}{\underset{\text{i}=1}{\sum }}{\left({\text{P}}_{\text{i}}-{\text{O}}_{\text{i}}\right)}^{2}}$$

**Table 1:** Summary of selected chlorophyll-related spectral indices reported in the literature.

Spectral indices	Formulation or depiction	Reference

RDVI	(R_800_-R_670_)/(SQRT(R_800_+R_670_))	Rougean and Breon, 1995
SR710	R_750/_R_710_	Zarco-Tejada et al., 2001
SR680	R_800_/R_680_	Jordan, 1969
RVI	R_810_/R_660_	Zhu et al., 2008
VOG 1	R_740_/R_720_	Vogelman et al., 1993
mND_705_	(R_750_−R_705_)/(R_750_+R_705_−2R_445_)	Sims and Gamon, 2002
PRI	(R_531_-R_570_)/(R_531_+R5_570_)	Gamon et al.,1992
GM	R_750_/R_550_	Gitelson and Merzlyak, 1996
MTCI	(R_750_-R_710_)/(R_710_-R_680_)	Dash and Curran, 2004
R-M	R_750_/R_720_-1	Sims and Gamon, 2002
NDRE	(R_790_−R_720_)/(R_790_+R_720_)	Barnes et al., 2000
MSR705	(R_750_-R_445_)/(R_705_-R_445_)	Sims and Gamon, 2002
MCARI2	((R_750_−R_705_)−0.2*(R_750_−R_550_))*(R_750_/R_705_)	Wu et al., 2008
OSAVI	1.16(R_800_-R_670_)/(R_800_+R_670_+0.16)	Rondeaux et al., 1996
OSAVI2	(1 + 0.16) * (R_750_ −R_705_)/(R_750_ + R_705_+ 0.16)	Wu et al., 2008
MSAVI	0.5*(2*R_800_+1−SQRT((2*R_800_+1)2−8* (R_800_−R_670_)))	Qi et al., 1994
TVI	0.5*(120*(R_750_−R_550_)−200*(R_670_−R_550_))	Broge and Leblanc, 2001
MTVI	1.2*(1.2*(R_800_-R_550_)-2.5*(R_670_-R_550_))	Haboudane et al., 2004
TCARI2/OSAVI2	TCARI2/OSAVI2	Wu et al., 2008
MCARI2/OSAVI2	MCARI2/OSAVI2	Wu et al., 2008
VI_opt_	(1 + 0.45)*((R_800_)^2^+1)/(R_670_+0.45)	Reyniers et al., 2006
CI green	R_NIR_/R_Red_-1	Gitelson, 2005
SPVI	0.4*3.7*(R_800_−R_670_)−1.2*SQRT((R_530_−R_670_)^2^)	Vincini et al., 2006
Datt2	R_850_/R_710_	Datt, 1999a
Datt3	D_754_/D_704_	Datt, 1999b
Gitel2	(R_750_−R_800_/R_695_−R_740_)−1	Gitelson et al., 2003
Voge	D_715_/D_705_	Vogelman et al., 1993
dSR	D_730_/D_706_	Zarco-Tejada et al., 2003
REArea	$\overset{680}{\underset{780}{\int^{\text{​}}}}\frac{{\text{dR}}_{\lambda }}{\text{d}\lambda }\text{d}\lambda$	Filella and Peñuelas, 1994
REA	Maximum value of first derivative in red-edge region	Filella and Peñuelas, 1994

R is the reflectance at the given wavelength. E.g., R_720_, R_740_ and R_750_ are the spectral reflectance at 720, 740 and 750nm, respectively; R*_λ_* is the spectral reflectance at wavelength *λ*; D*_λ_* denotes the first derivative value at wavelength *λ*.

Where P_i_ and O_i_ are predicted and observed CCC values and n is the number of samples.

## Results

### The First Derivative Spectra in the Red-Edge Region Under Different N Treatments

**Figure 6:**
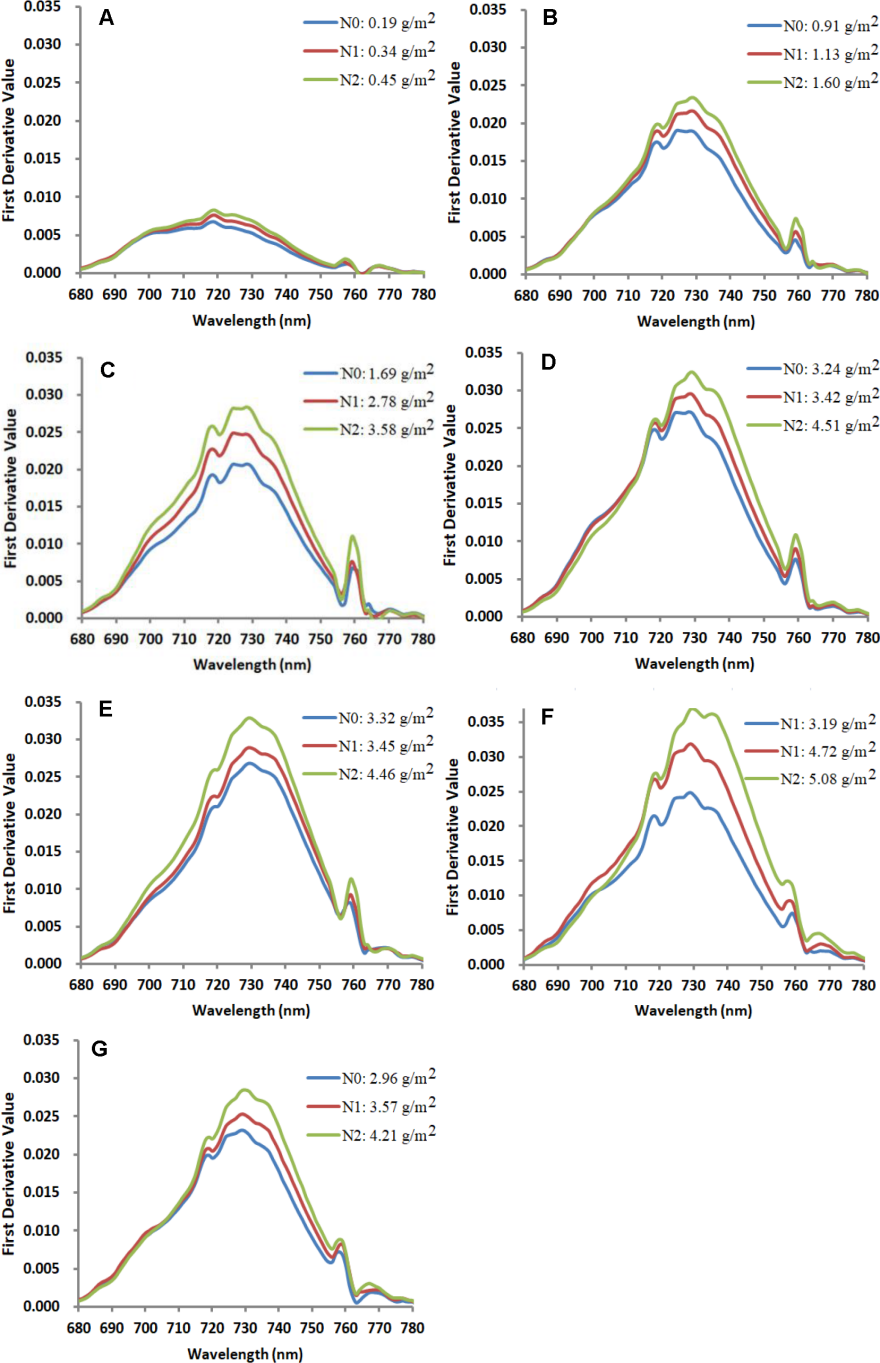
Response of first derivative spectra to nitrogen treatment at VE **(A)**, V6 **(B)**, V10 **(C)**, V12 **(D)**, VT **(E)**, R1 **(F)**, and R3 **(G)** growth stages in the Jingyu 7 cultivar in Exp.1. Note: each line stands for a single measurement. N0, N1, and N2 represent N application treatments of 0, 75 and 150 kg ha^−1^, respectively.

First derivative spectra of corn canopy in the red-edge region changed significantly with N treatments and CCC during different growth stages. **Figure 6** displays the response of first derivative spectra to N treatments in Jingyu 7 measured at different growth stages in Exp.1. The first derivative spectra of corn canopy were characterized by multiple peaks. The positions of the first peak, the second peak, and the third peak were always approximately at 718 nm, 729 nm and 759 nm, especially from the V6 to R3 (from **Figures 6B–G**) growth stages. Additionally, there was a clear minimum near 755 nm. It was found that the REArea and REA increased with an increasing amount of N fertilizer and CCC. However, REP was unstable due to the multiple peak phenomena. The REArea and REA showed an increasing trend from VE to R1 (from **Figures 6A–E**) growth stages. The REArea_760_ and REA_760_ were also highly positively correlated with CCC. The wavelength of the inflection point in the O_2_–A absorption band was stable near 759 nm. Moreover, the peak ranging from 755 nm to 763 nm was more blunt and the contrast between the minima near 755 nm and the peak near 759 nm reduced after VT growth stage (see **Figures 6F, G**).

#### Relationships Between CCC and Chlorophyll-Related Spectral Indices

**Figure 7:**
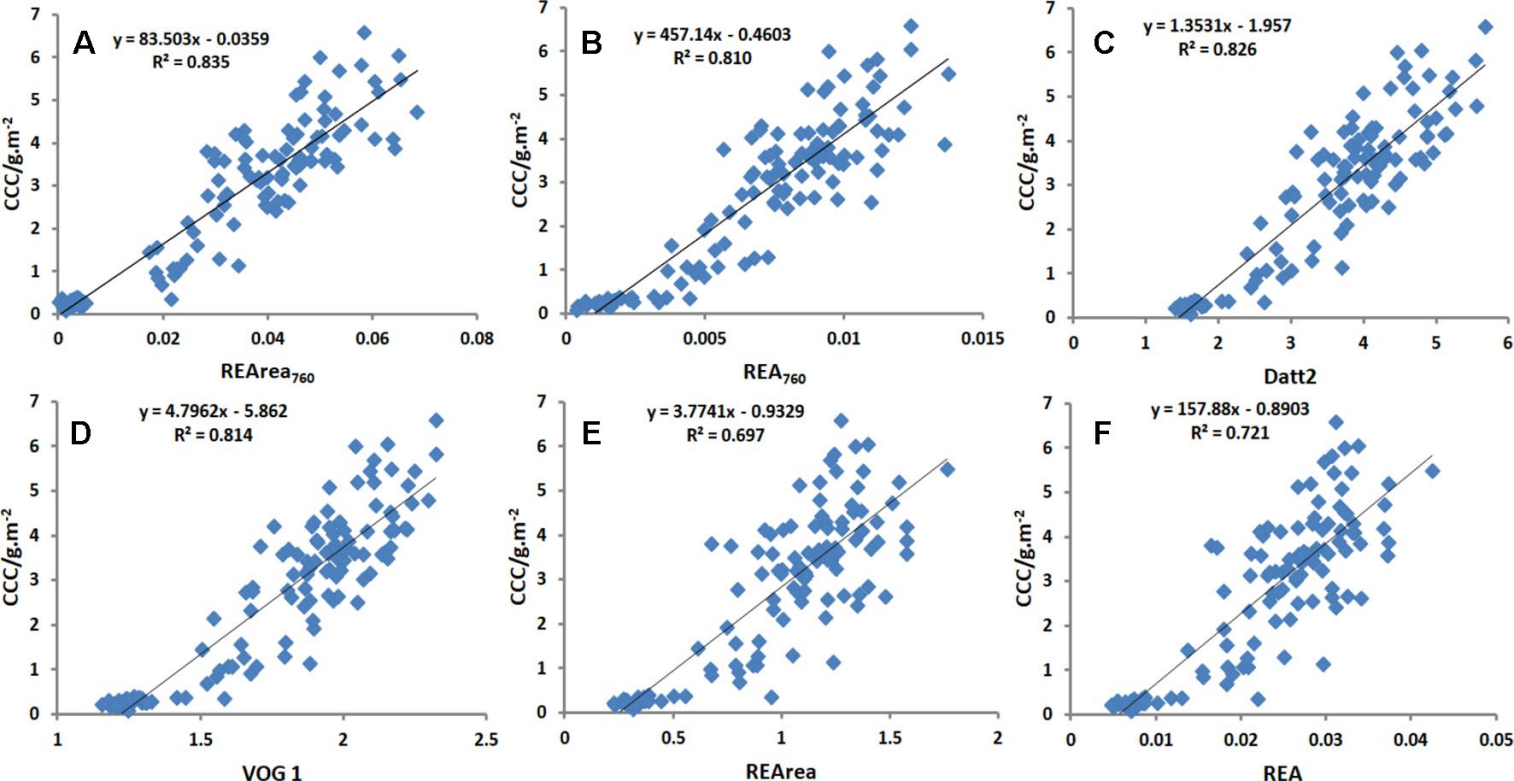
Linear relationship between CCC and spectral index [REArea 760, REA 760, Datt2, VOG 1, REArea and REA] for all cultivars in Exp.1. Among them REArea760 had the highest R2 value of 0.835 and REArea had the lowest R2 value of 0.697. REArea760 and REA760 were proved to be superior parameters, with high R2 values above 0.80.

**Table 2** shows that the linear relationships between CCC and 30 Chlorophyll-related spectral indices selected from literature and the two new spectral indices proposed in this study based on the observed data from Exp.1. Overall, the red-edge-related spectral indices were significantly correlated with CCC. R^2^ values ranged from 0.697 to 0.835, suggesting that spectral information in the red edge region was useful for assessing crop Chl status. As shown in **Table 2**, SR710,VOG1,GM, R-M, NDRE, and Datt2, as well as REArea_760_ and REA_760_, were proved to be superior parameters, with high R^2^ values above 0.80. In addition, compared with other commonly used red-edge-related spectral indices, the two novel red-edge parameters, especially REArea_760_, yielded relatively high R^2^ values for CCC. REArea_760_ performed better in estimating CCC than the traditional red edge parameters, such as REArea and REA, for which R^2^ values were 0.697 and 0.721. **Figure 7** displays the scatterplots between CCC and the spectral indices, REArea_760_, REA_760_, Datt2, VOG 1, REArea, and REA. Among them REArea_760_ had the highest R^2^ value of 0.835.

**Table 2:** Linear relationship between corn canopy chlorophyll content (CCC) and common red-edge spectral parameters using data from Exp.1. The ranking results of the performance (assessed using RMSE) of the 32 spectral indices to predict CCC using validation data from Exp.2. The spectral indices are ordered in ascending order according to their summed ranks.

Spectral indices	Linear equation	R^2^	Rank_ R^2^	RMSE (g/m^2)^	Rank_ RMSE	Summed rank

REArea_760_	y = 83.503x − 0.0359	0.835	1	0.663	5	6
VOG 1	y = 4.7962x − 5.862	0.814	5	0.655	3	8
Datt2	y = 1.3531x − 1.957	0.826	2	0.673	6	8
REA_760_	y = 457.14x − 0.4603	0.810	8	0.635	1	9
NDRE	y = 12.709x − 1.8291	0.805	9	0.647	2	11
SR710	y = 1.6178x − 2.2586	0.812	7	0.658	4	11
R-M	y = 3.4712x − 0.8742	0.824	3	0.675	8	11
Voge	y = 4.7962x − 5.862	0.814	6	0.756	14	20
MCARI2/OSAVI2	y = 2.6783x − 0.8115	0.799	10	0.754	13	23
OSAVI2	y = 10.625x − 1.8086	0.764	18	0.674	7	25
GM	y = 0.9933x − 2.0486	0.823	4	0.832	23	27
Datt3	y = 5.3196x − 0.2059	0.757	20	0.684	9	29
MCARI2	y = 3.9888x + 0.1518	0.792	11	0.823	22	33
OSAVI	y = 8.7403x − 2.2657	0.738	24	0.727	10	34
RDVI	y = 10.908x − 1.9155	0.739	23	0.745	12	35
MTCI	y = 1.2938x − 1.8484	0.73	26	0.732	11	37
MSAVI	y = 8.6642x − 1.1018	0.744	22	0.768	16	38
dSR	y = 2.5442x − 2.2088	0.752	21	0.782	17	38
MSR705	y = 0.796x − 1.3507	0.785	12	0.882	26	38
TCARI2/OSAVI2	y = -5.6499x + 3.8156	0.771	14	0.874	25	39
Gitel2	y = -0.5316x − 1.2055	0.785	13	0.972	28	41
REA	y = 157.88x − 0.8903	0.721	27	0.765	15	42
VI_opt_	y = 6.6255x − 18.754	0.767	15	0.961	27	42
mND_705_	y = 9.852x − 3.4495	0.735	25	0.786	18	43
SR680	y = 0.3321x − 0.2489	0.765	16	1.182	29	45
CI green	y = 0.3161x + 0.078	0.765	17	1.200	30	47
MTVI	y = 8.9571x − 0.7737	0.692	30	0.792	19	49
REArea	y = 3.7741x – 0.9329	0.697	29	0.810	20	49
RVI	y = 0.3416x − 0.2965	0.764	19	1.221	31	50
SPVI	y = 10.312x − 0.9526	0.717	28	0.835	24	52
TVI	y = 0.2478x − 0.8134	0.662	32	0.815	21	53
PRI	y = 63.75x + 4.5472	0.676	31	1.432	32	63

The number of pairs of data in Exp.1 was 120. y denotes canopy chlorophyll content (CCC) in corn, and x denotes spectral indices. R^2^ is the coefficient of determination.

#### Test of CCC Relationship to Chlorophyll-Related Spectral Indices

**Figure 8:**
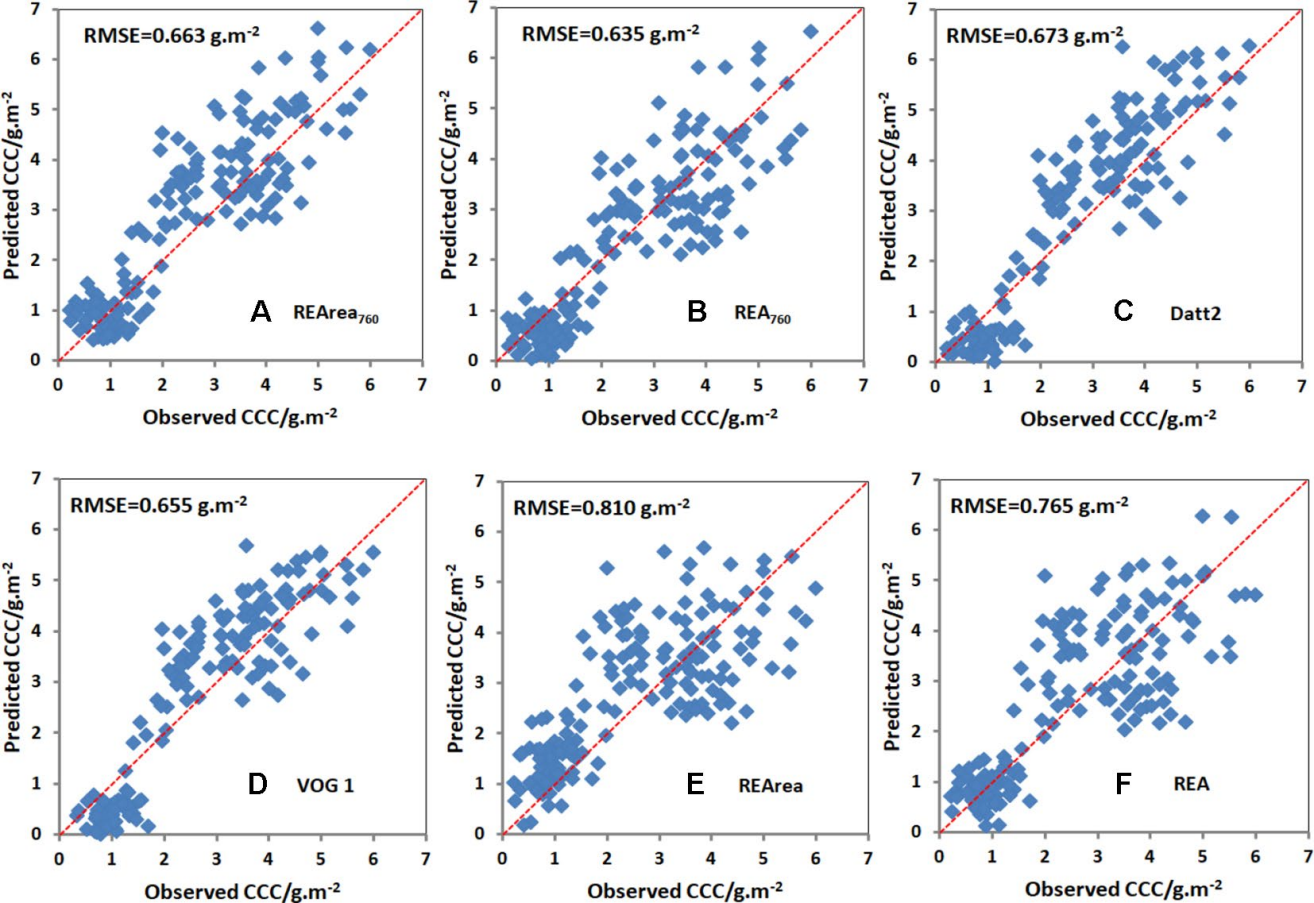
Comparison between the observed and predicted CCC based on REArea_760_ **(A)**, REA_760_ **(B)**, Datt2 **(C)**, VOG 1 **(D)**, REArea **(E)** and REA **(F)** for all cultivars in Exp.2. Note: The number of sample points used for validation was 178.

The relationships between CCC and the spectral indices described above were tested using data from Exp.2. RMSE was employed to measure the reliability and accuracy between estimated and observed values. As shown in **Table 2**, the estimation accuracy for REA_760_ has the lowest RMSE of 0.635 g/m^2^, and PRI has the highest RMSE value of 1.432 g/m^2^. **Figure 8** compares the observed and predicted CCC values generated from six spectral indices, including REArea_760_, REA_760_, Datt2, VOG 1, REArea, and REA. Their RMSEs were 0.663, 0.635, 0.673, 0.655, 0.810, and 0.765 g/m^2^, respectively. The model of REArea overestimated the CCC of corn when the CCC was low, e.g., less than 3 g/m^2^, and underestimated the CCC for high Chl content of canopy. The scatterplots of REArea (**Figure 8E**) and REA (**Figure 8F**) show more dispersion than those of REArea_760_ (**Figure 8A**), REA_760_ (**Figure 8B**), Datt2 (**Figure 8C**), and VOG 1 (**Figure 8D**), especially for high CCC. Their summed ranks were 6, 9, 8, 8, 49, and 42. Additionally, in **Table 2**, the indices were also sorted in ascending order according to their summed ranks by integrating the ranks of RMSE and R^2^ of CCC linear equation models. REArea_760_, Datt2, VOG 1, and REA_760_ ranked in the top four and the summed ranks were 6, 8, 8, and 9.

## Discussion

Similar to other crops, such as rice, cotton, soybean, and grass, the first derivative spectra of corn canopy were characterized by multiple peaks (see **Figure 6**) (Tang et al., 2004; Li et al., 2016). Previous research has shown that the derivative reflectance around the peaks was sensitive to plant Chl status, leaf area index, and canopy biomass (Yang et al., 2010). However, though the derivative method can minimize the influences of various linear signals, such as atmosphere and soil background, double-peak features of the vegetation derivative spectra weaken the usefulness of red edge parameters, such as REP in monitoring Chl content (Ju et al., 2010; Li et al., 2017). As showed in **Figure 4**, numerous previous studies provided additional evidence for the ChlF in-filling in O_2_−A band effects on apparent reflectance (Liu et al., 2005; Pérez-Priego et al., 2005; Ni et al., 2015). The changes of derivative values around 760 nm were not caused by the noise from strong water and oxygen absorption but indicated the changes on Chl content as well as ChlF (Ni et al., 2015) and biomass (Van der Tol et al., 2016).

Compared to the vegetative stages, the peak of first derivative spectrum in the O_2_−A band was not obvious during the reproductive stages (i.e., after R1 growth stage), and the contrast between the minima and peak of first derivative spectrum has gradually reduced. The possible reason is that nutrients (mainly nitrogen) were transfered from vegetative organs (e.g. shoots and leaves) to the reproductive organs (e.g. grain) (Salon et al., 2001), which led to the decreasing in leaf Chl content. Moreover, the tassel above the corn canopy also evidently contributed to the canopy reflectance spectrum after the reproductive stages.

As shown in this study, though the ChlF re-emitted by Chl molecules is very weak, F_760_ can represent the information on leaf Chl content, LAI, and biomass. Many previous studies also found that F_760_ was strongly correlated to LAI and biomass (Bånkestad and Wik, 2016; Van der Tol et al., 2016) and generally increased with increasing chlorophyll content (Buschmann, 2007). The fluorescent in-filling effects on reflectance results in the peak of the first derivative spectra in the O_2_−A band, and two indices—REArea_760_ and REA_760_—were proposed to estimate CCC of corn canopy in this study.

The summed ranks of REArea and REA were far higher than those of REArea_760_ and REA_760_ (**Table 2**). The possible reason is that double-peak feature has little effect on the derivative spectral characteristics in the O_2_−A band. Moreover, the performance of REArea_760_ and REA_760_ was also better than most of the reflectance-based indices selected from previous studies. ChlF originates only from chlorophyll in the photosynthetic apparatus and materials such as soil, wood, and dead biomass also absorb PAR but do not contribute to photosynthesis. ChlF therefore is less sensitive to soil, wood, and dead biomass interference (Daughtry et al., 2000; Porcar-Castell et al., 2014; Bånkestad and Wik, 2016). Therefore, it can improve the accuracy of CCC estimation.

A simplified formulation has also been used to express steady state ChlF (Joiner et al., 2014), i.e.,

(7) $$\text{SIF}\left(\text{t},\lambda \right)=\text{PAR}\left(\text{t}\right)\times \text{fPAR}\left(\text{t}\right)\times \Theta_{\text{F}}\left(\text{t},\lambda \right)\times \text{e}\left(\text{t},\lambda \right)$$

where SIF is solar-induced ChlF, λ is the excitation wavelength, PAR is the incident photosynthetically active radiation, fPAR is the fraction of photosynthetically active radiation, Θ_F_ is the fluorescence efficiency and e is the fractional amount of fluorescence that escapes the canopy. However, SIF is emitted by the canopy colony (Liu and Chen, 2011), and many factors would impact it, such as PAR, fPAR, Θ_F_ and e (Equation 7) The diurnal variation trend in the SIF is similar to that of PAR (Liu et al., 2015, Liu et al., 2017). REArea_760_ and REA_760_ were derived from the reflectance spectrum and therefore the influence of PAR on the two indices can be largely reduced. fPAR and e were determined by canopy structure and vegetation biochemistry (Joiner et al., 2014). In other words, the two parameters contain the information about canopy structure and vegetation biochemistry. The red- but not far-red fluorescence was readily reabsorbed (Bånkestad and Wik, 2016). Therefore, the reabsorption of far-red SIF, e.g. F_760_, caused by complex canopy structure or increasing leaf chlorophyll content was much smaller than for the red bands and is usually neglected (Van Wittenberghe et al., 2015). The e parameter is considered to be less important in crop and grass canopies (Damm et al., 2015; Liu et al., 2017).

The fluorescence yield of PSI is generally low and invariant to illumination change (Franck et al., 2002; Porcar-Castell et al., 2014). The contribution from PSI fluorescence has a higher relative contribution at longer wavelengths since PSI fluorescence peaks at longer wavelengths than PSII fluorescence. However, the fluorescence yield of PSI is typically much smaller than that of PSII. Therefore, fluorescence at 760 nm is still dominated by variable fluorescence from PSII (Porcar-Castell et al., 2014). The number of PSII reaction centers is known to increase with irradiance (Anderson et al., 1988). Thus, the fluorescence yield at 760 nm is influenced by light intensity. The spectral data in the paper were measured from the same species on sunny days in summer, which probably caused the relatively stable fluorescence yield due to little changes in the measurement conditions, e.g. light intensity. This may be one of the important reasons that the fluorescence-based indices proposed in this study function fairly well.

In addition, the method developed in the paper has been tested with only one species and by using point measurements. In order to improve reliability and robustness of the method, more species and approaches to data acquisition, e.g. imaging measurement, should be taken into account in future research.

## Conclusions

Timely assessment of CCC in crops is critical for diagnosing the growth stage, maximizing yield, and minimizing adverse environmental impacts. In this study, combining ChlF signals in the O_2_–A absorption band, an *in situ* hyperspectral remote sensor was used to estimate corn Chl content at the canopy level. Two new spectral indices, REArea_760_ and REA_760_, were proposed because the double-peak feature of vegetation derivative spectra weakens the usefulness of red edge parameters in monitoring CCC. REArea_760_ and REA_760_ models were compared with existing red edge indices as well as REArea and REA and proved stable and powerful enough for monitoring CCC. The novel parameters for CCC estimation were proved to be accurate for corn under different environmental conditions and across many typical growing seasons. Even so, further validation is needed to test the stability and the robustness of the two indices by considering canopy phenological stages and transfer ability of the spectral indices in estimating CCC for other crops.

## Data Availability

All datasets for this study are included in the manuscript and the supplementary files.

## Author Contributions

XZ and GW conceived and designed the research. CT, CW, FX, YH, and XL analyzed the data. XZ and DC wrote the manuscript. CT, GW, and LS assisted manuscript writing and editing.

## Funding

The data of the paper were from the spectral database system of typical objects in China. This work was supported by the National Natural Science Foundation of China (41871239 and 61601229), China Postdoctoral Science Foundation (2017M610338 and 2019M650125), the Open Fund of Key Laboratory of Meteorology, Ecological Environment of Hebei Province (Z201607Y), National Key Research and Development Program of China (2018YFD0300805), Priority Academic Program Development of Jiangsu Higher Education Institutions (PAPD), Hebei Innovation Capability Promotion Project(18964201H), and the Natural Science Foundation of Jiangsu Province (BK20160966).

## Conflict of Interest Statement

The authors declare that the research was conducted in the absence of any commercial or financial relationships that could be construed as a potential conflict of interest.
